# Prehistoric migrations through the Mediterranean basin shaped Corsican Y-chromosome diversity

**DOI:** 10.1371/journal.pone.0200641

**Published:** 2018-08-01

**Authors:** Julie Di Cristofaro, Stéphane Mazières, Audrey Tous, Cornelia Di Gaetano, Alice A. Lin, Paul Nebbia, Alberto Piazza, Roy J. King, Peter Underhill, Jacques Chiaroni

**Affiliations:** 1 Etablissement Français du Sang PACA Corse, Biologie des Groupes Sanguins, Marseille, France; 2 Aix Marseille Univ, CNRS, EFS, ADES, "Biologie des Groupes Sanguins", Marseille, France; 3 Department of Medical Sciences, University of Turin, Turin, Italy; 4 Department of Psychiatry, Stanford University School of Medicine, Stanford, California, United States of America; 5 Sartène Museum, Sartène, France; 6 Department of Genetics, Stanford University School of Medicine, Stanford, California, United States of America; Banaras Hindu University, INDIA

## Abstract

The rarity of human remains makes it difficult to apprehend the first settlements in Corsica. It is admitted that initial colonization could have occurred during the Mesolithic period when glaciations would have shortened the open water travel distance from the continent. Mesolithic sites in Corsica show relatively short and irregular occupation, and suggest discontinuous settling of very mobile groups probably traveling by boat. Previous genetic studies on Corsican populations showed internal differentiation and a relatively poor genetic relationship with continental populations, despite intense historical contacts, however local Mesolithic-based genetic inheritance has never been properly estimated. The aim of this study was to explore the Corsican genetic profile of Y-chromosomes in order to trace the genetic signatures back to the first migrations to Corsica. This study included 321 samples from men throughout Corsica; samples from Provence and Tuscany were added to the cohort. All samples were typed for 92 Y-SNPs, and Y-STRs were also analyzed. Results revealed highly differentiated haplogroup patterns among Corsican populations. Haplogroup G had the highest frequency in Corsica, mostly displaying a unique Y-STR profile. When compared with Provence and Tuscany, Corsican populations displayed limited genetic proximity. Corsican populations present a remarkable Y-chromosome genetic mixture. Although the Corsican Y-chromosome profile shows similarities with both Provence and to a lesser extent Tuscany, it mainly displays its own specificity. This study confirms the high level of genetic diversity in Corsican populations and backs genetic contributions from prehistoric migrations associated with the Mesolithic, Neolithic and Metal Age eras, rather than from historical movements to Corsica, respectively attested by frequencies and TMRCA of haplogroups G2a-L91 and G2a-P15, J2a-M241 and J2-DYS445 = 6, R1b-U152 and R1b-U106. These results suggest that marine routes to reach the Corsican coast in many different points may have led to such a genetic heterogeneity.

## Introduction

Corsica is the fourth largest island of the Mediterranean, located 177km south-east of Provence and 85km west of Tuscany. The rarity of human remains due to the acidity of natural sediments, the sea-level rising and uncertainty regarding the Paleolithic era, all make it difficult to apprehend the first settlements in Corsica.

The most widely accepted hypothesis concerning Homo-Sapiens Corsican settlements is the colonization of the Corsico-Sardinian block via Tuscany throughout the different glacial periods and occupation of the south of the block (currently Sardinia) followed by that of the north (Corsica). The initial colonization may have occurred during the Mesolithic period (18–15,000 years ago) [1]. The most ancient presence of humans is attested by the Mesolithic collective burial site of Campo Stefano (South of Corsica) dated at circa 8,940 BP [2]. Other Mesolithic sites are located in southwestern (Filitosa River) and southern Corsica as well as in Sardinia [2–5]. Major demographic changes occurred in Corsica mainly during the Neolithic period, from the 6^th^ millenium BC onwards, due to external contributions attested by an importation of foreign stones to the island, flint and obsidian, and Impressed Ware, Cardial and Bell-Beaker potteries [4,6,7] (S1 Table). Corsican prehistory ended when the Greeks built the city of Alalia in 565 BCE. Following a long occupation by the Romans, from 269 BCE, the island underwent several waves of consecutive invasions (by the Vandals, Byzantines and the Roman papacy) [8].

Surname-based studies showed a very low rate of exogamy between the different Corsican micro-regions, especially in the north and far south of the island [9,10]. Corsican genetic studies on autosomal markers and the Y-chromosome confirmed this differentiation between the south and the rest of the island [10–12].

Genetic relationships between Corsica and Sardinia are controversial. Mitochondrial and Y-chromosome studies showed almost no gene flow transmitted by Sardinian men to Corsica or continental Italy [6,12,13]. More particularly, the I-M26 haplogroup which is rare or absent anywhere other than Sardinia, but homogeneously present throughout this island, is the proof of a founder effect [14]. Conversely, other studies found that the Sardinian population seemed to have a high genetic affinity with the Corsicans [11,15,16].

On a Mediterranean scale, genetic studies showed two clusters: one in the west, including north-west and central Corsica and the south of Sardinia, and one with central and northern Sardinia, southern Corsica, Sicily and Turkey [10,17]. On the contrary, some studies found that current Corsican populations were genetically distinct from Mediterranean populations [18] and that continental populations from Tuscany and France did not seem to have significantly contributed towards the genetic structure of the Corsican populations [19,20] which rather reflected contributions from Southern Europe [18].

Thus, contradictory results have been published concerning the Corsican genetic composition and the relationship with Sardinia and other neighboring regions such as France and Tuscany. It seems clear however that there is a high rate of heterogeneity within the Corsican population, especially between the North and South, supported by archaeological, anthropological and linguistic data. Continental contributions to the Corsican populations seem slight despite intensive historical interactions.

The aim of this study is to explore the genetic profile of a sample of the Corsican population in order to retrace the genetic signatures back to the first migrations to the island during prehistoric times. This study explores the paternal side of the population, including 321 male samples throughout Corsica which were genealogically characterized. Genetic analyses of the Y-chromosome were carried out, part of which has already been published [21,22]. In order to place our samples in a Mediterranean context, samples from Provence (France) [22] and Tuscany (Italy) were added to the cohort. Finally, Y-chromosome results were compared to databases from published literature on Mediterranean populations.

## Material and methods

### Sampling

A total of 841 individuals from Corsica (France), Provence (France) and Tuscany (Italy) were analyzed. Sample names, geographical coordinates and sample sizes are detailed in Table 1. All samples were collected from healthy donors. All participants signed an informed consent in agreement with the guidelines of the ethical committee of the institutions involved. The study protocol was approved by the *Ministère de l’Enseignement Supérieur et de la Recherche (Ministry of Higher Education and Research)* in France (record number DC-2008-164, formally approved on December 15th, 2008 by the French Ministry of Research).

**Table 1:** Description of the 18 Corsican, 1 Provençal and 3 Tuscan populations under study.

Populations	Micro-regions	Lat.	Long.	Total (N)
CORSICA	Bastia	42.6972	9.45088	31
	Cap	42.7328	9.36609	8
	Saint Florent	42.6818	9.30370	7
	Balagne	42.6121	8.89077	17
	Calvi	42.5676	8.75722	4
	Casinca	42.4671	9.45996	26
	Ponte Leccia	42.4635	9.20707	14
	Campuloru	42.3159	9.54729	12
	Travu	42.5277	9.35638	22
	Bravone	42.1977	9.55611	7
	Corte	42.3094	9.14902	27
	Sevi	42.2234	8.83009	24
	Taravo	41.9353	9.15521	26
	Ajaccio	41.9192	8.73863	42
	Alta Rocca	41.7282	9.21205	28
	Extreme South	41.7340	9.33455	14
	Valinco	41.7168	8.91761	7
	Sartene	41.6218	8.97471	5
**Total CORSICA**				**321**
**PROVENCE**		**43.2964**	**5.36977**	**259**
TUSCANY	Siena	43.1668	11.3658	86
	Arezzo	43.6985	11.8194	62
	Pisa	43.3993	10.8660	113
**Total TUSCANY**				**261**

DNA was extracted from whole blood using the Qiamp blood kit (Qiagen, Courtaboeuf, France). The populations surveyed were either evaluated for the first time in this study, or updated to higher levels of phylogenetic resolution than reported in earlier studies [21,22].

### Y-chromosome SNP analyses

All samples were typed for 92 Y-SNPs. First, 19 SNPs of the main haplogroups were typed in a multiplex SnapShot (Applied Biosystems, France) protocol described in S2 Table: A-P97, B-M181, C-M130, D-M174, E-M96, F-M89, G-M201, H-M69, I-M258, J-M304, K-M526, L-M11, M-Page93, N-M231, O-M175, Q-M242, R-M207, S-M230 and T-M70. Secondly, genotyping was designed to refine each main lineage previously identified. Haplogroups R, J and G were genotyped by specific SNaPshot multiplex protocols. Three multiplex assays were developed for respectively 4, 9 and 4 SNPs of the R sub-haplogroups (Multiplex assay for haplogroup R analysis number 1: M9, SRY1532, M449 and M198; Multiplex assay for haplogroup R analysis number 2: M479, M124, M343, M269, M412, M207, M173, P297 and M478; and Multiplex assay for haplogroup R analysis number 3: S116, M529, U152 and M405); 2 multiplex assays for 3 and 13 SNPs of the sub-haplogroups J (Multiplex assay for haplogroup J analysis number 1: M304, M267 and M172; Multiplex assay for haplogroup J analysis number 2: L222, M530, M304, M267, M92, Page8, M205, M12, M322, Page55, M340, M172, M410 and M67) and 1 multiplex assay for 11 SNPs of the G sub-haplogroups (P15, M406, M201, M377, M547, M285, M286, M287, P16, M527 and P303). SNaPshot multiplex assays were performed according to the manufacturer’s protocol and recommendations. PCR and extension primers and their concentrations are given in S2 Table. Y Haplogroups or sub-haplogroups were analyzed using GeneMapper® 4.0.

Further derived samples were genotyped in a hierarchical manner for the 31 markers by direct sequencing (Big Dye 1.1 according to the manufacturer’s protocol): haplogroup C: PK2, haplogroup E: M33, V38, M215, M35, M78, V13, V22, M81, M123, M34, haplogroup G: L91, PF3147, L497, M377, haplogroup I: M72, M253, M227, M438, P37.2, M26, M423, M436, M223, haplogroup J: M241, haplogroup Q: M346, M378, haplogroup R: L23, U152, L11 and V88.

Genomic specifications for all markers have been previously reported [23–25] or listed on ISOGG (http://www.isogg.org/tree/).

Corsican samples had been previously typed for M201, P287, P15 and L91 [21] and for V13 [26]. Samples from Provence had been previously typed for E-V13, M406, Page94, M423, M269 and the following J-lineages: M304, Page55, M267, M12, M410, M67, M530, M92 and DYS445 [26].

### Y-STR based phylogenetic networks

Samples derived for haplogroups DYS445 = 6, L497, L91, M12, M241, M267, M406, M410, M527, M530, M67, M92, M96, P15, P16, P287, P303, Page55, Page8, V13 and V22, i. e. 298 samples, were typed for short tandem repeats of 17 Y-STR markers using the AmpFlSTR Yfiler Kit (Applied Biosystems) according to manufacturer recommendations. Three additional markers, DYS388, DYS445 and DYS461, were typed separately (S3 Table) [27,28].

Phylogenetic networks were constructed for haplogroups represented by more than 10 individuals using the program Network 4.6.1.1 (Fluxus-Engineering) and applying the median joining algorithm with the following STR loci: DYS19, DYS388, DYS389I, DYS389II, DYS390, DYS391, DYS392, DYS393, DYS439 [29]. Networks were constructed both at a Corsican level and at a broader level with data from Provence (this study and [22]), Tuscany and continental Italy [30].

### Time to the Most Recent Common Ancestor (TMRCA)

TMRCA for haplogroups with frequencies above 3% were estimated using eight Y-STR markers (DYS19, DYS388, DYS389I, DYS389II, DYS390, DYS391, DYS392 and DYS393) and a mean evolutionary rate mutation of 0.000069 ±0.000013 per locus per 25 years [31].

### Spatial frequency maps

Spatial frequency maps were drawn up for haplogroups and related sub-haplogroups that were detected with a frequency of at least 3%. Frequency data were converted into frequency maps using Surfer software (version 8, Golden Software, Inc.), following the *kriging* interpolation method [32].

### Mediterranean genetic relationships

Y chromosome haplogroup frequencies from 83 Mediterranean populations in published data (North Africa, Iberia, Italian Peninsula, Adriatic Balkans and Western Mediterranean islands) totalizing 7588 men were harmonized to a common set of 12 lineages: E-M96 (xM35), E-M35 (xM78), E-M78, G-M201, I-M170, J1-M267, J2-M172, R2-M479, R1-M173, K-LT-NOP-M9 (xM207), CF-M130-M89 (xM201, xM170, xM304, xM9), and Y(xM174, xM130, xM89) [6,14,33–42]. Genetic relationships within the Liguria and Tyrrhenian Seas were analyzed in a subset of 31 populations out of 83 refined for 23 haplogroups: E-M96 (xM78), E-M78, E-M81, G-M201, J1-M267, J2-M172*, J2-M12, J2-M410 (xM67, xDYS445 = 6), J2-DYS445 = 6, J2-M67*, J2-M92, R2-M124, R1a-M173*, R1a1-M17, R1b-M269, I (xM438), I2-M438 (xM26, xM423), I2-M26, I2-M423, I2-M223, Q-M242, Y (xM96, xM522, xM201), and K-M9 (xM74). Y-Chromosome genetic diversity based on haplogroup frequencies was calculated with the ARLEQUIN v3.5.1.2 package [43]. Finally, our Y-STR data was merged with those from Sardinia, Sicily and continental Italy [30]. Variance and Rst genetic distances were respectively plotted through a Principal Component analysis (PCA) and Multidimensional Scaling (MDS) using the XLSTAT tool (Data Analysis and Statistical Solution for Microsoft Excel. Addinsoft, Paris, France, 2017).

## Results

### Corsican genetic structure and diversity

**Fig 1:**
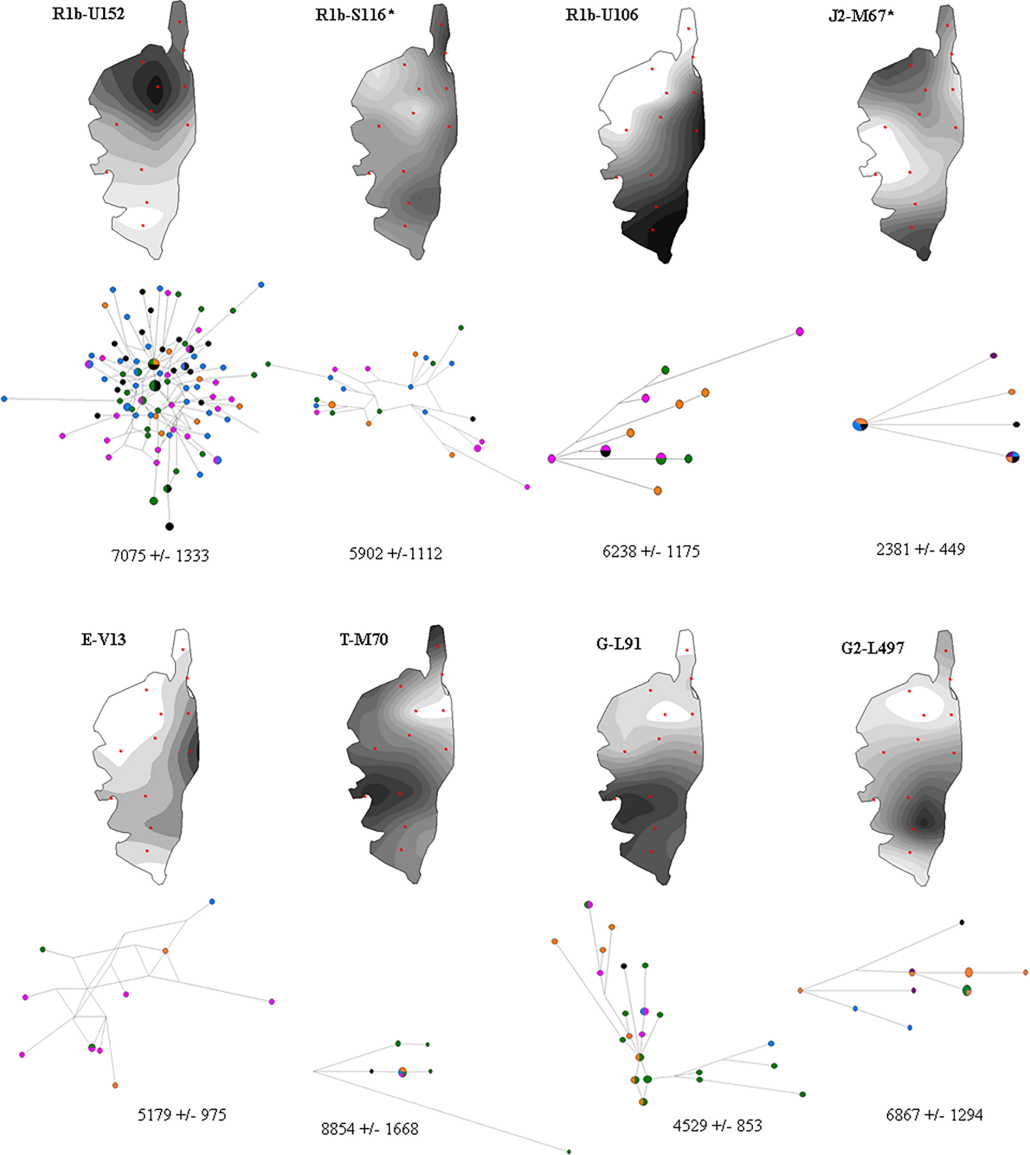
Spatial frequency maps for haplogroups with frequencies above 3%, their Y-STR based phylogenetic networks in Corsican populations (Blue: North, Green: West, Orange: South, Black: Center and Purple: East) and their TMRCA (in years, +/- SE).

Hierarchic phylogenetic relationships and frequencies, as well as haplogroup diversity of the 54 paternal haplogroups observed in the 321 Corsican, 259 Provencal and 261 Tuscan samples are described in S1 Fig. Spatial frequency maps performed on haplogroups with frequencies above 3%, their Y-STR based phylogenetic networks and TMRCA are presented in Fig 1.

Haplogroup R represented approximately half of the lineages in both Corsican and Tuscan samples (respectively 51.8% and 45.3%) whereas it reached 90% in Provence. Sub-clade R1b1a1a2a1a2b-U152 predominated in North Corsica whereas R1b1a1a2a1a1-U106 was present in South Corsica. Both SNPs display clinal distributions of frequency variation in Europe, the U152 branch being most frequent in Switzerland, Italy, France and Western Poland. Calibrated branch lengths from whole Y chromosome sequencing [44,45] and ancient DNA studies [46] both indicated that R1a and R1b diversification began relatively recently, about 5 Kya, consistent with Bronze Age and Copper Age demographic expansion. TMRCA estimations are concordant with such expansion in Corsica.

Haplogroup G reached 21.7% in Corsica and 13.3% in Tuscany. Sub-clade G2a2a1a2-L91 accounted for 11.3% of all haplogroups in Corsica yet was not present in Provence or in Tuscany. Thirty-four out of the 37 G2a2a1a2-L91 displayed a unique Y-STR profile, illustrated by the star-like profile of STR networks (Fig 1). G2a2a1a2-L91 and G2a2a-PF3147(xL91xM286) show their highest frequency in present day Sardinia and southern Corsica compared to low levels from Caucasus to Southern Europe, encompassing the Near and Middle East [21,47–50]. Ancient DNA results from Early and Middle Neolithic samples reported the presence of haplogroup G2a-P15 [51–53], consistent with gene flow from the Mediterranean region during the Neolithic transition. Td expansion time estimated by STR for P15-affiliated chromosomes was estimated to be 15,082+/-2217 years ago [49]. Ötzi, the 5,300-year-old Alpine mummy, was derived for the L91 SNP [21]. A genetic relationship between G haplogroups from Corsica and Sardinia is further supported by DYS19 duplication, reported in North Sardinia [14], and observed in the southern part of the Corsica in 9 out of 37 G2a2a1a2-L91 chromosomes and in 4 out of 5 G2a2a-PF3147(xL91xM286) chromosomes, 3 of which displayed an identical STR profile (S4 Table). This lineage has a reported coalescent age estimated by whole sequencing in Sardinian samples of about 9,000 years ago. This could reflect common ancestors coming from the Caucasus and moving westward during the Neolithic period [48], whereas their continental counterparts would have been replaced by rapidly expanding populations associated with the Bronze Age [46,54,55]. Estimated TMRCA for L91 lineage in Corsica is 4529 +/- 853 years. G-L497 showed high frequencies in Corsica compared to Provence and Tuscany, and this haplogroup was common in Europe, but rare in Greece, Anatolia and the Middle East. Fifteen out of the 17 Corsican G2a2b2a1a1b-L497 displayed a unique Y-STR profile (S4 Table) with an estimated TMRCA of 6867 +/- 1294 years. Haplogroup G2a2b1-M406, associated with Impressed Ware Neolithic markers, along with J2a1-DYS445 = 6 and J2a1b1-M92 [22,49], had very low levels in Corsica. Conversely, G2a2b2a-P303was highly represented and seemed to be independent of the G2a2b1-M406 marker. The 7 G2a2b2a-P303(xL497xM527) Corsican chromosomes displayed a unique Y-STR profile (S4 Table).

Haplogroup J, mainly represented by J2a1b-M67(xM92), displayed intermediate frequencies in Corsica compared to Tuscany and Provence. J2a1b-M67(xM92) derived STR network analysis displayed a quite homogeneous profile across the island with an estimated TMRCA of 2381 +/- 449 years (Fig 1) and individuals displaying M67 were peripheral compared to Northwestern Italians (S2 Fig). The haplogroup J2a1-Page55(xM67xM530), characteristic of non-Greek Anatolia [22], was found in the north-west of Corsica. Haplogroup J2a1-DYS445 = 6 was found in the north-west with DYS391 = 10 repeats, and in the far south with DYS391 = 9 repeats, the former was associated with Anatolian Greek samples, whereas the second was found in central Anatolia [22]. The 7 J2b2a-M241 displayed a unique Y-STR profile (S4 Table), they were only detected in the Cap Corse region, this sub-haplogroup shows frequency peaks in both the southern Balkans and northern-central Italy [56] and is associated with expansion from the Near East to the Balkans during Neolithic period [57].

Haplogroup E, mainly represented by E1b1b1a1b1a-V13, displayed intermediate frequencies in Corsica compared to Tuscany and Provence. E1b1b1a1b1a-V13 was thought to have initiated a pan-Mediterranean expansion 7,000 years ago starting from the Balkans [52] and its dispersal to the northern shore of the Mediterranean basin is consistent with the Greek Anatolian expansion to the western Mediterranean [22], characteristic of the region surrounding Alaria, and consistent with the TMRCA estimated in Corsica for this haplogroup. A few E1b1a-V38 chromosomes are also observed in the same regions as V13.

Haplogroup I was not found in Provence whereas it was present in both Corsica and Tuscany, respectively as I1 and I2 lineage, although at low levels. Haplogroup I is widespread throughout Europe with a strong geographic differentiation but virtually absent elsewhere. Haplogroup I1 is mostly found in northern Europe; haplogroup I2 has two sub-branches defined by P37.2 and M436 markers, respectively. The latter sub-branch is widespread Balkan Peninsula [58]. Ancient DNA results from Neolithic samples reported the presence of haplogroups I2-M438 and I2a1-P37.2 in southern France [52]. The I2a1a1-M26 marker, found in 30% of Sardinian samples [14,48], was present at very low levels in Corsica.

Haplogroup Q-M242 was mostly found in Sevi (5 of the 6 chromosomes) with quite similar STR patterns (S4 Table). Haplogroup Q, reported to have originated in Central Asia, is distributed widely in North Eurasia and at low frequencies in Europe, East Asia, and the Middle East [59].

Haplogroup T1a-M70, known as Thomas Jefferson’s Y chromosome and relatively rare in other Near Eastern populations [60], was observed in Corsica with an estimated TMRCA of 8854 +/- 1668 years.

### Mediterranean populations’ genetic relationships

The Corsican population displayed a significant portion of variance when compared with Provence and Tuscany (Fst = 11.57%, p = 0.000). The greatest variance was found when four groups were made up (Fst = 12.11%, p = 0.000) with Pisa, north-western Corsica (Corte, Balagne-Calvi and Ponte Leccia), Provence, and the rest of Corsica, labeled East/South Corsica.

**Fig 2:**
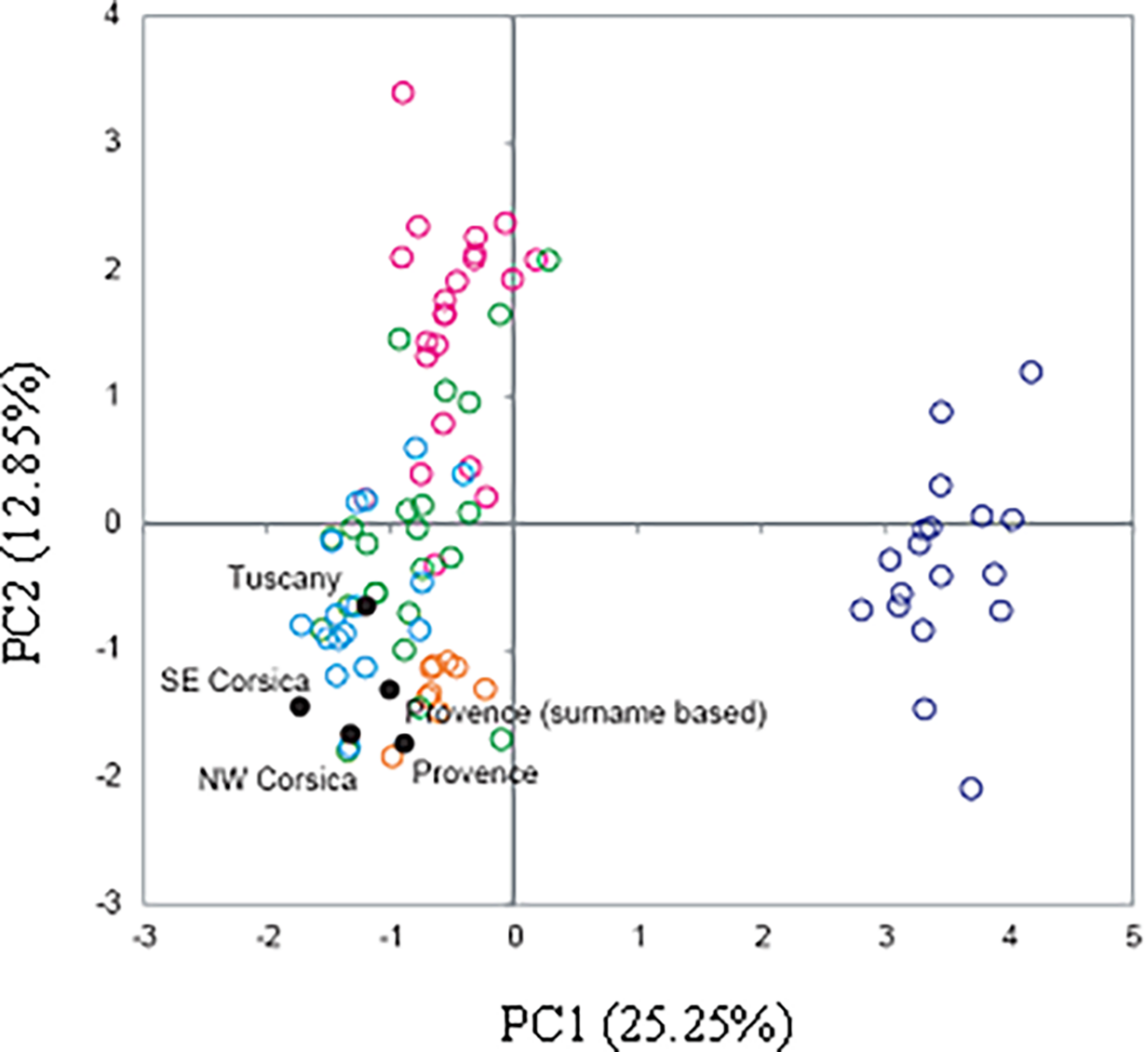
First and second axes of the PCA based on 12 Y-chromosome haplogroup frequencies in 83 west Mediterranean populations. North Africa: Purple, Balkans: Pink, Iberia: Orange, Continental Italy: Blue, West Mediterranean Islands: Green.

PCA (Fig 2) shows the genetic relationships across the western Mediterranean basin with Corsican populations presented according to SAMOVA results (SAMOVA results not shown). PC1 and 2 account for 38% of total variance. PC1 shows strong departure of Balkan, Iberian, Italian and insular populations from North Africa, due to the exclusive J1-M267 and E-M35 (xM78) in the latter. PC2 shows an east-to-west dispersal of the continental populations along the northern rim of the Mediterranean basin. In this continental pattern, the Tuscan samples under study cluster with those from continental Italy, whereas those from Provence are placed between samples from Iberia and continental Italy. Western Mediterranean islands Sicily and Sardinia cover all continental populations from the Balkans to Italy. North-west Corsican populations coincide with North-West Apulia (Italy) [34] and with Ibiza, whereas East/South Corsican populations are the furthest away from all of the western Mediterranean populations.

Haplogroup frequency analysis supports genetic similarities between Corsican, Cagliari and northern Sardinian Tempio populations, whereas Tuscan and Provencal samples cluster with continental Italy. Y-STR data showed genetic relationships of Provence and northern Corsican samples with Tuscan, central Italian and Ligurian populations, while southern Corsicans clustered with southern Italians and Sicily (S3 and S4 Figs).

## Discussion

The genetic profile of Corsican Y-chromosomes was explored in order to retrace the genetic signatures back to the first migrations to the island during prehistoric times. A total of 842 individuals including 321 from Corsica, 259 from Provence (France) and 261 from Tuscany (Italy) were analyzed for Y-chromosome haplogroups. Their results were compared to databases from published literature on Mediterranean populations.

Our results showed a highly heterogeneous distribution of Y-chromosome haplogroups in the 183km long and 83km-wide island, with a mosaic of numerous small-scale, micro-regional population groups.

The most ancient Corsican genetic signatures might be represented by haplogroup I2 derived individuals, as this haplogroup was reported to be associated to human expansion phase of Sardinia during pre-Neolithic period [14]. Neolithic migrations to Corsica from the Caucasus moving westward may be supported by the high frequency of haplogroup G-L91 and its estimated TMRCA [21,47–49]. The Alta Rocca region is well known for its archaeological records, particularly in Curacchiaghiu, attesting human presence during the early Neolithic (5650 BCE) and late Neolithic (2980 years BCE) periods [4,61]. Early Neolithic culture in Curacchiaghiu may have encountered two trends, one restricted in space and time, characterized by “pseudo-cardial” pottery, and one, characterizing most of early Neolithic remains, with stamped-impressed pottery. Notably, this region is characterized by a concentrated G-L497 frequency with a dispersed STR profile and an estimated TMRCA concordant with the Neolithic period. Our results show that L91-G2a and L497-G2a lineages are quite unique to the Corsican population. This might be a signature of Neolithic migration conserved over time due to particular Corsican geography and a low rate of exogamy whereas on the Continent they were replaced by expansion during the Bronze Age [21,46]. Haplogroups associated to Neolithic Impressed Ware (J-DYS445 = 6 and J-M92) [22,49] found in the north and south of the island were respectively associated to STR described in Anatolian Greek samples and central Anatolia which further supported independent settlements on the island during the Neolithic period. Furthermore, the haplogroup J-M12, only detected in the Cap Corse region and described to correlate with the distribution of archaeological painted pottery and anthropomorphic figurines [26] suggested an additional Neolithic migration route.

Differential distribution of R1b-U152 and R1b-U106 haplogroups and their respective TMRCA seem to coincide with the two groups of Menhir-statue mostly erected during the Bronze Age. The northern Menhir-statue group has slim figures and prominent ears, whereas in the larger Menhir-statue group, located south of the Ajaccio-Solenzara line, they are rougher and display warrior attributes [4,5,61].

Greek Anatolian historical settlement in Alaria coincided strikingly with haplogroup E-V13 distribution [22]. Haplogroup Q-M242 is mostly represented by sub-group M346 in the Sevi area. M346 appears in Central, Western and Southern Asia, and most parts of Europe [62].

These results support the hypothesis of multiple discontinuous settling in Corsica and may hint a presence of Mesolithic lineages in Corsican patrilineal gene pool, in accordance with archaeological data from Mesolithic, Neolithic and Bronze Age periods. They also confirmed, as previously reported by genetic and surname-based studies, a very low rate of exogamy within the island leading to Y-chromosome pattern distribution structured by geography, due to the mountainous landscape of Corsica.

Concerning genetic relationships between Corsica and Sardinia, our results corroborated with different genetic heritage for both islands. However, high genetic affinity between the Sardinian population and the Corsicans was previously reported [11,15,16] and is supported by the similarity of the southern Corsican and Sardinian Mesolithic sites [2,3], as well as the similarity of the Corsican « Torrean » and Sardinian « Nuragic » cultures. Our results on haplogroup G samples and their STR profile are in accordance with a genetic continuity between South Corsica and Sardinia [14]. Conversely, our results for haplogroup I support a genetic distinction between these two islands as previously reported [6,12,13].

The Corsico-Sardinian block is characterized by climatic contrast: glacial sediments found in the north of Corsica reveal three glacial periods, whereas these sediments are absent in Sardinia where the climate was characterized by rainy periods. It is reasonable to assume that the first groups of humans would have preferentially settled in regions where the climate was more clement. Then, only when the climate became warmer, would these populations have moved towards Corsica from the north of Sardinia [16]. Overall, archaeological and genetic data support a common Mesolithic genetic background, estimated in Sardinia at ~7700 years ago [47] and transformed by Neolithic migrations [63] and Bronze Age heritage [4,5,61].

On a western Mediterranean scale, genetic studies have published controversial results, with studies showing on the one hand affinities between southern Corsica, central and northern Sardinia, Sicily and Turkey, and on the other hand between north-west and central Corsica and the south of Sardinia [10,17]; on the contrary, other studies found that current Corsican populations were genetically distinct from other Mediterranean populations [18–20].

Our results support genetic affinities between north-western Corsica and continental populations whereas East/South Corsican populations originated from western Mediterranean populations. These two clusters are in accordance with the dramatic divergence of populations from north and south Corsica described here and supported by genetic, linguistic and archaeological studies and possibly explained by a divergence of population history accumulated since Neolithic times between the north and south of the island. These results are in accordance with the hypothesis of different genetic destinies for populations from mainland Europe and those from the islands of the Tyrrhenian sea, reported to have begun to diverge at least 5,000 years ago [21].

In conclusion, whereas it is widely believed that settlers in Corsica arrived from mainland Italy, due to a difference in the sea level during glaciations and the formation of a natural bridge with Tuscany, our results support that Corsica might have been colonized by many different waves of migration since Neolithic period and Bronze Age and led to such a heterogeneous Y-chromosome profile of the current Corsican population. The overall complexity of the genetic profile of Corsicans remains to be fully addressed with the study of complementary mitochondrial and autosomal markers.

## Supporting information

S1 Fig Corsica, Provence and Tuscany Y-chromosome tree. Hierarchic phylogenetic relationships and frequencies (percentages), haplogroup diversity observed in the 321 Corsican, 259 Provencal and 261 Tuscan samples.
(XLSX)

S2 Fig Y-STR based phylogenetic networks. Populations from Corsica, Provence and Tuscany under study and Italian populations from Boattini A, et al. 2013 [30].
(TIF)

S3 Fig First and second axes of the PCA based on 23 Y-chromosome haplogroup frequencies in 31 populations from the northern rim of the Mediterranean basin. (TIF)

S4 Fig Multidimensional Scaling (MDS) of Rst genetic distances between populations of the Tyrrhenian Sea (stress: 0.659). Black: present study, open squares: Oltremontano speakers, filled squares: Cismontano speakers. Others colors refer to the eight Italian areas in Boattini A, et al. 2013 [30] (Light Green: Northwestern Italy, Grey: Northeastern Italy, Red: Bologna, Pink: Tuscany, Light Blue: Central Italy, Yellow: Southern Italy, Blue: Sicily, Green: Sardinia).
(TIF)

S1 Table Chronological List of Major Archaeological Strata adapted from D'Anna A et al. 2007 [61]. (DOCX)

S2 Table Primers and their concentrations used for multiplex SNaPshot assays to simultaneously analyze: main Y-chromosome haplogroups, haplogroups R (3 multiplex analyses), haplogroup J (2 multiplex analyses) and haplogroup G. F and R stand for Forward and Reverse respectively.
(XLSX)

S3 Table Primers and their concentrations used for multiplex fragment analysis assay to simultaneously analyze STR DYS388, DYS445 and DYS461. F and R stand for Forward and Reverse respectively.
(DOC)

S4 Table Y-STR data from this study, Boattini et al. 2013 and King et al. 2011. (XLSX)

## Abbreviations

kya: Thousand years ago
BCE: Before Christ Era
SNP: Single Nucleotide Polymorphism
Y-STR: Y-chromosome Short Tandem Repeat
TMRCA: Time to the Most Recent Common Ancestor
