# Supplementary figures and images for: Prehistoric migrations through the Mediterranean basin shaped Corsican Y-chromosome diversity
Source: PLoS One. 2018 Aug 1;13(8):e0200641. doi: 10.1371/journal.pone.0200641 (PMC6070208; doi:10.1371/journal.pone.0200641)

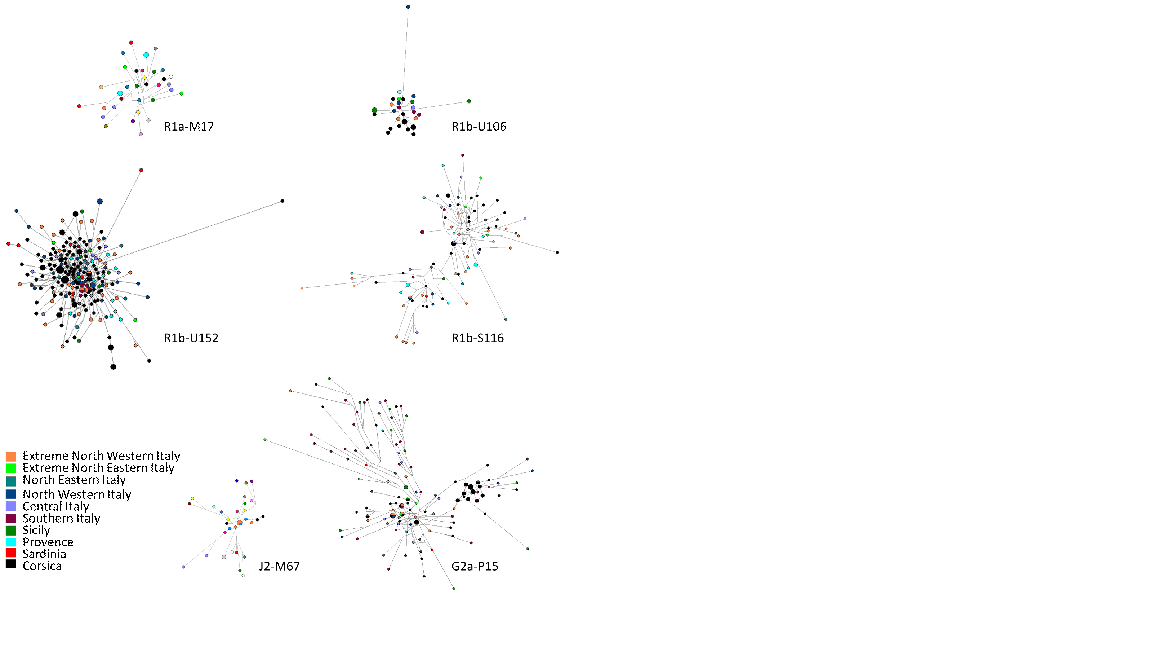

Supplement: S2 Fig — Populations from Corsica, Provence and Tuscany under study and Italian populations from Boattini A, et al. 2013 [30]. (TIF) [file pone.0200641.s002.tif]

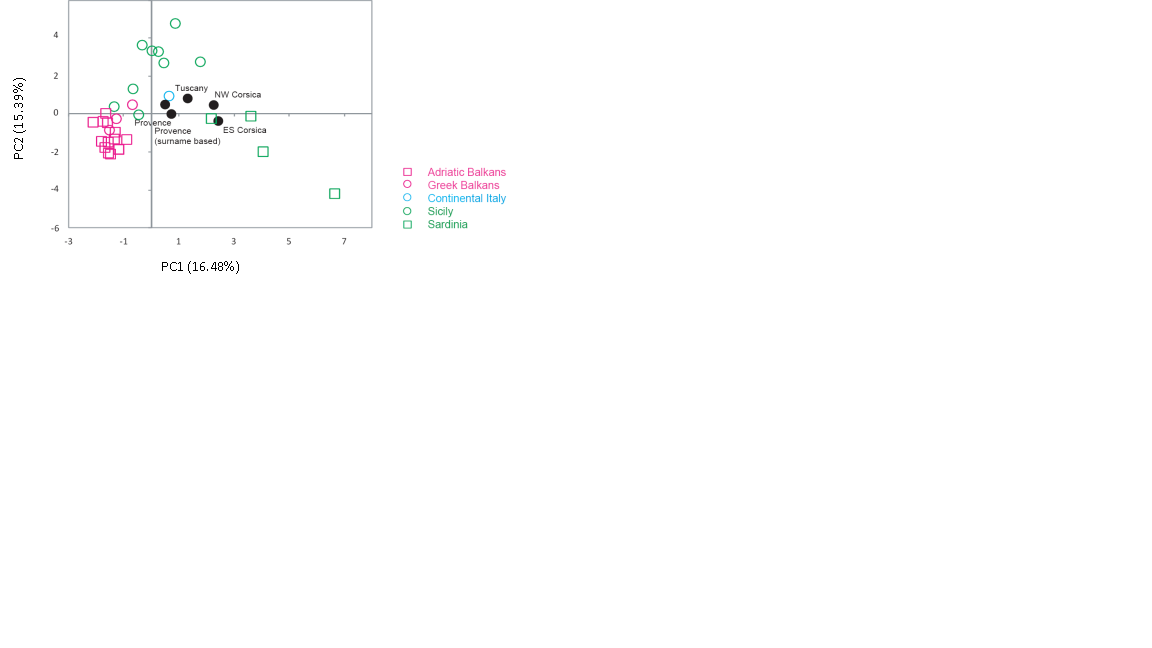

Supplement: S3 Fig — (TIF) [file pone.0200641.s003.tif]

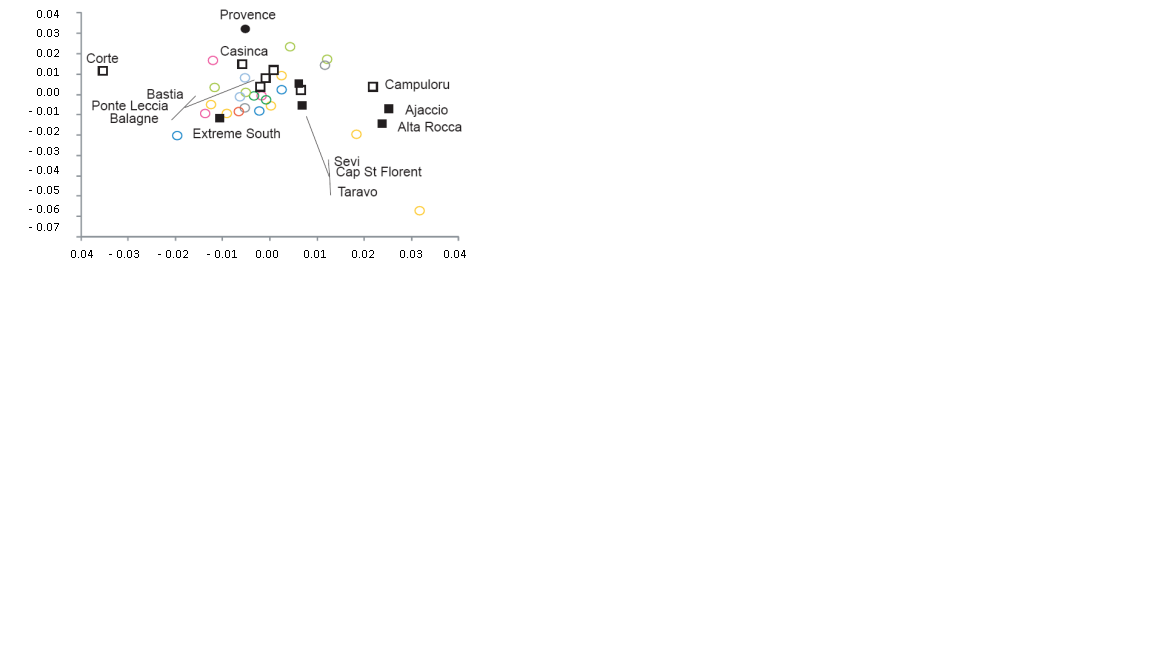

Supplement: S4 Fig — Black: present study, open squares: Oltremontano speakers, filled squares: Cismontano speakers. Others colors refer to the eight Italian areas in Boattini A, et al. 2013 [30] (Light Green: Northwestern Italy, Grey: Northeastern Italy, Red: Bologna, Pink: Tuscany, Light Blue: Central Italy, Yellow: Southern Italy, Blue: Sicily, Green: Sardinia). (TIF) [file pone.0200641.s004.tif]
