# Supplementary material for: Prehistoric migrations through the Mediterranean basin shaped Corsican Y-chromosome diversity
Source: PLoS One. 2018 Aug 1;13(8):e0200641. doi: 10.1371/journal.pone.0200641 (PMC6070208; doi:10.1371/journal.pone.0200641)
Supplement: S1 Table — (DOCX) [file pone.0200641.s005.docx]

**Table S1: Chronological List of Major Archaeological Strata adapted from D'Anna A *et al.* 2007 [61].**

| Period | Dates BP | Sites and Characteristics |
| --- | --- | --- |
| ***Fauna of continental origin*** | | |
| Pleistocene | 350,000 | Kastric network, Nebbiu |
| ***Neanderthal*** | | |
| Pleistocene | 70,000-60,000 | La Coscia cave, Rogliano, Castiglione pit, Oletta |
| ***Homo Sapiens*** | | |
| Mesolithic | 8,940 | Campo Stefano, Sollacaro |
| Mesolithic | 8,300 | Monte Leone, Bonifacio |
| Mesolithic | Holocene | Nomads - Coastal Fishermen |
| Imported-Continental Neolithic | 8,000 | Initial colonization by non-natives  Dramatic Shift :Breeding - Feral animals, Silex, Obsidian, Cardial pottery, Impressed pottery, |
| Insular specific Neolithic | 7,000-6,000 | Evolution in subsidiary mode: Bovine breeding, Dairy feeding, Sardinian obsidian |
| Chalcolithic- Early Bronze | 5,500-4,000 | Terrinean culture, Megalithic, Campaniform pottery, House Forms, modest metalworking activity |
| Bronze Age | 4,000-2,800 | Torrean I, II and III cultures, nurargic-style habitats |
