# Supplementary material for: Prehistoric migrations through the Mediterranean basin shaped Corsican Y-chromosome diversity
Source: PLoS One. 2018 Aug 1;13(8):e0200641. doi: 10.1371/journal.pone.0200641 (PMC6070208; doi:10.1371/journal.pone.0200641)
Supplement: S3 Table — F and R stand for Forward and Reverse respectively. (DOC) [file pone.0200641.s007.doc]

**Table S3. Primers and their concentrations** used for multiplex fragment analysis assay to simultaneously analyze STR DYS388, DYS445 and DYS461. F and R stand for Forward and Reverse respectively.

| STR | PCR Primer Sequence | | Final concentration (µM) in the multiplex PCR | Amplicon size according to repetition number |
| --- | --- | --- | --- | --- |
| DYS388 | F | VIC-gtgagttagccgtttagcga | 0.2 | 143pb for 17 ATT |
|  | R | cagatcgcaaccactgcgct | 0.2 |  |
| DYS461 | F | NED-aggcagaggatagatgatatggat | 1.2 | 182pb for 11 TAGA or for 10 GATA |
|  | R | ttcaggtaaatctgtccagtagtg | 1.2 |  |
| DYS445 | F | 6-FAM-agttaagagccccaccttcctg | 0.2 | 263pb for 12 TTTA |
|  | R | gagctgagattatgccaccaaaa | 0.2 |  |
